# HASTF: a hybrid attention spatio-temporal feature fusion network for EEG emotion recognition

**DOI:** 10.3389/fnins.2024.1479570

**Published:** 2024-10-14

**Authors:** Fangzhou Hu, Fei Wang, Jinying Bi, Zida An, Chao Chen, Gangguo Qu, Shuai Han

**Affiliations:** ^1^Faculty of Robot Science and Engineering, Northeastern University, Shenyang, China; ^2^Department of Neurosurgery, Shengjing Hospital of China Medical University, Shenyang, China

**Keywords:** affective Brain-Computer Interface, EEG, emotion recognition, hybrid attention, spatio-temporal feature

## Abstract

**Introduction:**

EEG-based emotion recognition has gradually become a new research direction, known as affective Brain-Computer Interface (aBCI), which has huge application potential in human-computer interaction and neuroscience. However, how to extract spatio-temporal fusion features from complex EEG signals and build learning method with high recognition accuracy and strong interpretability is still challenging.

**Methods:**

In this paper, we propose a hybrid attention spatio-temporal feature fusion network for EEG-based emotion recognition. First, we designed a spatial attention feature extractor capable of merging shallow and deep features to extract spatial information and adaptively select crucial features under different emotional states. Then, the temporal feature extractor based on the multi-head attention mechanism is integrated to perform spatio-temporal feature fusion to achieve emotion recognition. Finally, we visualize the extracted spatial attention features using feature maps, further analyzing key channels corresponding to different emotions and subjects.

**Results:**

Our method outperforms the current state-of-the-art methods on two public datasets, SEED and DEAP. The recognition accuracy are 99.12% ± 1.25% (SEED), 98.93% ± 1.45% (DEAP-arousal), and 98.57% ± 2.60% (DEAP-valence). We also conduct ablation experiments, using statistical methods to analyze the impact of each module on the final result. The spatial attention features reveal that emotion-related neural patterns indeed exist, which is consistent with conclusions in the field of neurology.

**Discussion:**

The experimental results show that our method can effectively extract and fuse spatial and temporal information. It has excellent recognition performance, and also possesses strong robustness, performing stably across different datasets and experimental environments for emotion recognition.

## 1 Introduction

Emotion is a complex psychological and physiological state in human life, typically manifested as subjective feelings, physiological responses, and behavioral changes (Maithri et al., 2022). Emotions can be brief or enduring, significantly influencing individual and social interactions. The recognition of emotions can enhance human-machine interactions and improves user experiences, meeting user needs more effectively. It also aids in diagnosing and treating mental health issues such as Autism Spectrum Disorder (ASD) (Zhang et al., 2022) and Bipolar disorder (BD) (Zhao et al., 2024). Compared to non-physiological signals like facial expressions (Ge et al., 2022), speech and text (Hung and Alias, 2023), or body posture (Xu et al., 2022), physiological signals offer higher reliability, are less susceptible to deception, and provide more objective information. Physiological tests like Electroencephalogram (EEG) (Su et al., 2022), Electromyography(EMG) (Ezzameli and Mahersia, 2023), Electrocardiogram (ECG) (Wang et al., 2023), and Electrooculogram (EOG) (Cai et al., 2023), are commonly used. Among them, EEG signals are commonly used as they record the electrical activity of brain neurons and provide direct information about emotional processing. Additionally, EEG signals are acquired non-invasively, at low cost and offers very high temporal resolution.

In the past researches, in order to further improve the recognition accuracy of emotion and uncover brain responses during the emotion induction process, considerable research efforts have been devoted to extracting and classifying emotional features of EEG signals. EEG signals contain a wealth of information. Extracting appropriate features not only reduces data dimensionality and complexity but helps emphasize the crucial information related to emotions as well. In 2013, Duan et al. (2013) proposed Differential Entropy(DE) features for emotion recognition and achieved high recognition accuracy. And in 2017, Zheng et al. (2017) extracted PSD, DE, DASM and other features, inputting them into the same classifiers for emotion classification. They found DE can improve the accuracy the most compared to the baseline. Gong et al. (2023) also found that extracting DE features would achieve higher accuracy. Therefore, in this study we also extracted DE features. For classifiers, commonly used machine learning classifiers mainly include Support Vector Machine (SVM) (Mohammadi et al., 2017), Decision Tree (DT) (Keumala et al., 2022), K Nearest Neighbor (KNN) (Li M. et al., 2018), Random Forest (RF), etc. However, with the rapid development of deep learning methods, their adaptability and excellent performance have made them increasingly popular for emotion recognition. In 2015, Zheng and Lu (2015) designed a Deep Belief Network (DBN), results show that the DBN models outperform over other models with higher mean accuracy and lower standard deviations. Chao et al. (2023) designed ResGAT based on graph attention network, it greatly improved accuracy compared to traditional machine learning. In Fan et al. (2024), the accuracy of LResCapsule proposed by Fan et al. is 10–20% higher than that of DT and SVM.

Recently, more researchers have focused on utilizing deep learning to extract information from different domains within EEG signals, aiming to obtain more comprehensive information.

In the spatial domain, extracted features can reflect relationships between EEG channels. Some researchers treat each channel as a node, introduce topological structure, and employ Graph Neural Networks (GNN) to capture the relationships between channels. For example, Song et al. (2018) proposed a dynamic graph convolutional neural network (DGCNN) for multi-channel EEG emotion recognition, which extracts intrinsic relationships among EEG channels. Similarly, Ye et al. (2022) and Li et al. (2024) also based their work on GCN, using hierarchical and multi-branch approaches to obtain richer spatial information. While this approach simulates connectivity relationships among EEG channels, GCNs typically use information from first-order neighboring nodes for graph convolution, focusing on the nearest nodes and potentially ignoring more distant ones. For EEG data, channel relationships do not necessarily correlate with physical proximity. Another approach is to map EEG channels to a 2D matrix based on their original layout, and process them similarly to image data (Yang et al., 2018a; Shen et al., 2020; Li J. et al., 2018; Xiao et al., 2022). However, the resulting matrix does not match typical image sizes, and each “pixel” represents an EEG channel, which is not equivalent to pixels in an image (Xu et al., 2024). Consequently, applying image processing techniques can lead to information loss. Therefore, the primary issue to consider is how to effectively utilize the spatial domain information in EEG signals and construct deep learning networks that align with this structure to extract information accurately.

Emotional states are dynamic, and EEG signals are acquired in a time sequence, requiring consideration of their temporal dynamics. Xing et al. (2019) employed LSTM-RNN to capture the temporal information of emotions, using contextual correlation to improve classification accuracy. Si et al. (2023) proposed MATCN, which extracts local temporal information through separable convolution with attention and captures global temporal information through a Transformer, demonstrating its potential in temporal emotional localization. While domain-specific features of EEG signals exhibit distinct characteristics, they are not entirely isolated; complementary and redundant information exists between them. Some researchers combine different deep learning networks to learn features from various domains (Pan et al., 2023; Cheng et al., 2024; Li D. et al., 2022; Wei et al., 2024; Gong et al., 2024; Sartipi et al., 2023). Specifically, Li D. et al. (2022) introduced STGATE, a composite framework, which employs a Transformer network to learn time-frequency representations and Graph Attention Network (GAT) to learn spatial representations. Sartipi et al. (2023) proposed a hybrid structure combining a spatio-temporal encoder with a recurrent attention network to learn spatio-temporal features. Fusing features from different domains can effectively utilize complementary information and eliminate redundancy, significantly enhancing emotion recognition accuracy. Fully integrating spatio-temporal feature information after effectively extracting spatial domain information is the second issue to consider.

Emotions are complex, arising from the brain's biological mechanisms. Researchers in the neurological field strive to understand these mechanisms to provide deeper insights into the treatment of emotional disorders (McRae et al., 2012; Buhle et al., 2014; Messina et al., 2021). This study goes beyond simply categorizing emotions. It further investigates how emotions are generated at the neural level and decodes their traces in the brain (Bo et al., 2024). Therefore, the final issue to consider is conducting an in-depth analysis of EEG signals. We aim to capture activity levels and neural oscillation patterns in various brain regions to reveal the spatio-temporal dynamics of emotion processing, and visualize common neural patterns corresponding to different emotional states and subjects.

Thus, in summary, we have identified the following research problems: (a). How to design spatial feature extractors that align with EEG structure, mitigate information loss, and ensure precise information extraction. (b). How to integrate spatio-temporal features by leveraging complementary information and reducing redundancy. (c). How to apply engineering methods to capture brain activity across regions, revealing neural patterns linked to different emotional states and subjects.

To address the above problems, this paper proposes a Hybrid Attention Spatio-Temporal Feature Fusion Network (HASTF). HASTF contains a spatial attention feature extractor and a temporal attention feature extractor. It extracts more discriminative spatial information, adaptively selecting crucial regions and electrode channels. The temporal attention feature extractor uses a multi-head attention mechanism to extract global temporal feature information, and fuse it with spatial features. While completing an emotion recognition task, it also visualize the activity levels of various brain regions during the generation of different emotions.

The primary contribution of this study can be summarized as:

(1) We apply a parameter-free attention to the spatial feature extraction network for the first time, which can directly calculate attention weights for the 3D feature. And we add skip connections to solve the problem of information loss, selecting crucial channels related to emotions adaptively by learning and capturing more fine-grained spatial features.

(2) We design HASTF, a hybrid attention model. It can effectively utilizes the advantages of spatial domain attention and temporal domain attention to fuse the spatial and temporal features of EEG signals. HASTF fully leverages the complementary nature of spatio-temporal features, enhancing the accuracy of emotion recognition. We tested it on two public datasets, achieved state-of-the-art performance.

(3) While achieving high recognition accuracy, we leverage the strengths of HASTF to visualize the activation states of brain regions corresponding to different emotions and different subjects, exploring the neural patterns of emotions in the brain from an engineering perspective.

The rest of paper is divided into five sections as follows: Section 2 provides a detailed elaboration of proposed model. Section 3 introduces the two publicly datasets and provides a comprehensive report on various experimental setups conducted on these datasets. Section 4 summarizes the experimental results and conducts an analysis of these findings. Sections 5, 6, respectively, discuss and conclude the research work of this paper.

## 2 Methods

**Figure 1:**
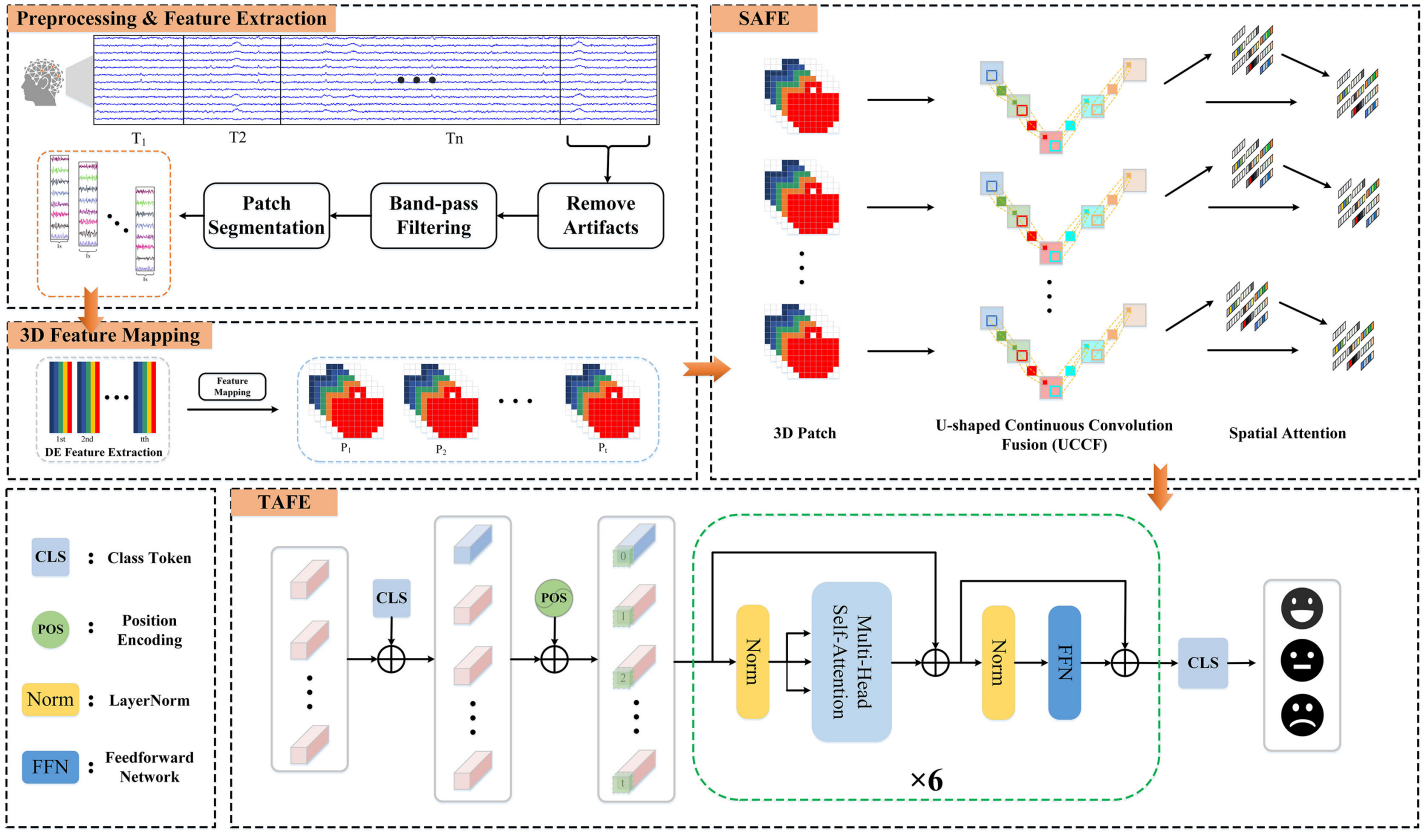
The framework diagram of the hybrid attention spatio-temporal feature fusion network (HASTF) for EEG emotion recognition.

In this section, we introduce the methods and the Hybrid Attention Spatio-Temporal Feature Fusion Network (HASTF) proposed in this paper. As shown in Figure 1, the overall framework can be divided into four parts: Preprocessing & Feature Extraction, 3D Feature Mapping, Spatial Attention Feature Extractor (SAFE) and Temporal Attention Feature Extractor (TAFE). Each part will be introduced in detail below.

### 2.1 Preprocessing and feature extraction

For the preprocessed EEG signal *X* ∈ ℝ^(*C*×*L*)^, where C represents the number of EEG channels and L is the EEG sampling time. According to Cai et al. (2024) and Yi et al. (2024), EEG contains various frequency components, which are associated with different functional states of the brain. Therefore, we first use a third-order Butterworth bandpass filter to decomposed the signal into Delta (1–4 Hz), Theta (4–8 Hz), Alpha (8–13 Hz), Beta (13–31 Hz), and Gamma (31–50 Hz), which can be described as Equation 1. This ensures a smooth transition at frequency boundaries and effective separation.


(1) $$\begin{array}{l} \left\{ \begin{array}{l} y_{\text{Delta}}=\text{butter\_pass}\left(1,4\right)\\ y_{\text{Theta}}=\text{butter\_pass}\left(4,8\right)\\ y_{\text{Alpha}}=\text{butter\_pass}\left(8,13\right)\\ y_{\text{Beta}}=\text{butter\_pass}\left(13,31\right)\\ y_{\text{Gamma}}=\text{butter\_pass}\left(31,50\right) \end{array}\right. \end{array}$$

Then, the signal is non-overlappingly segmented into n time windows of length T. In this study, T is set to 8 s for the DEAP dataset and 11 s for the SEED dataset. Each time window is further divided into smaller patches of 1-s length. For each patch, DE features are extracted in the five frequency bands according to the following formula:


(2) $$\begin{array}{l} DE\left(X\right)=\int_{-\infty }^{\infty }f\left(x\right)\log f\left(x\right)dx \end{array}$$

Since the EEG signals within each subband tend to closely approximate a Gaussian distribution, f(x) can be written as $f\left(x\right)=\frac{1}{\sqrt{2\pi \sigma^{2}}}\exp\left(-\frac{{\left(x-\mu \right)}^{2}}{2\sigma^{2}}\right)$. Where μ and σ^2^ represent the expectation and variance of X, respectively. Therefore, the result of the above formula is


(3) $$\begin{array}{l} DE\left(X\right)=\frac{1}{2}\log\left(2\pi e\sigma^{2}\right) \end{array}$$

Here, e is the base of the natural logarithm.

### 2.2 3D feature mapping

**Figure 2:**
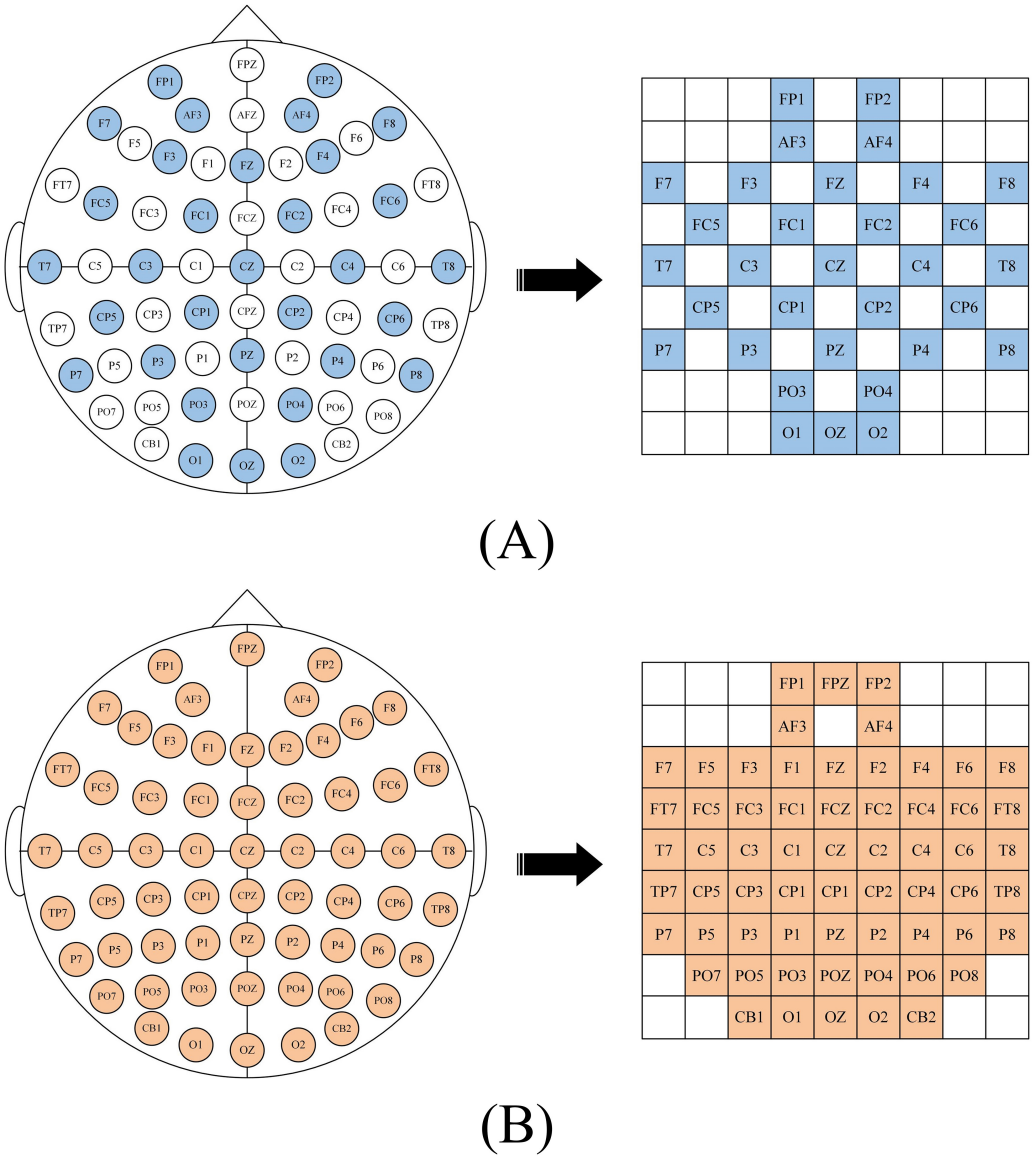
2D mapping of **(A)** DEAP dataset, **(B)** SEED dataset.

After calculating the DE characteristics of each frequency band, we process the EEG data into patches one by one. Each patch *P* ∈ ℝ^(*n*×*T*×*B*×*C*)^, where n is the number of time windows, T is the number of patches in each time window, B is the number of frequency bands, here B = 5, C is the number of channels. When collecting EEG, electrode caps that comply with the international 10-20 standard are used for data collection. What we want is to restore the arrangement of electrodes on the brain and integrate frequency, spatial and temporal characteristics. Specifically, we arrange the electrode channels in a 2D matrix format that preserves their relative positions as they are placed on the scalp (Yang et al., 2018a; Shen et al., 2020; Xiao et al., 2022). Figure 2 is the mapping diagram of 32 and 62 electrode channels.

### 2.3 Spatial attention feature extractor

**Figure 3:**
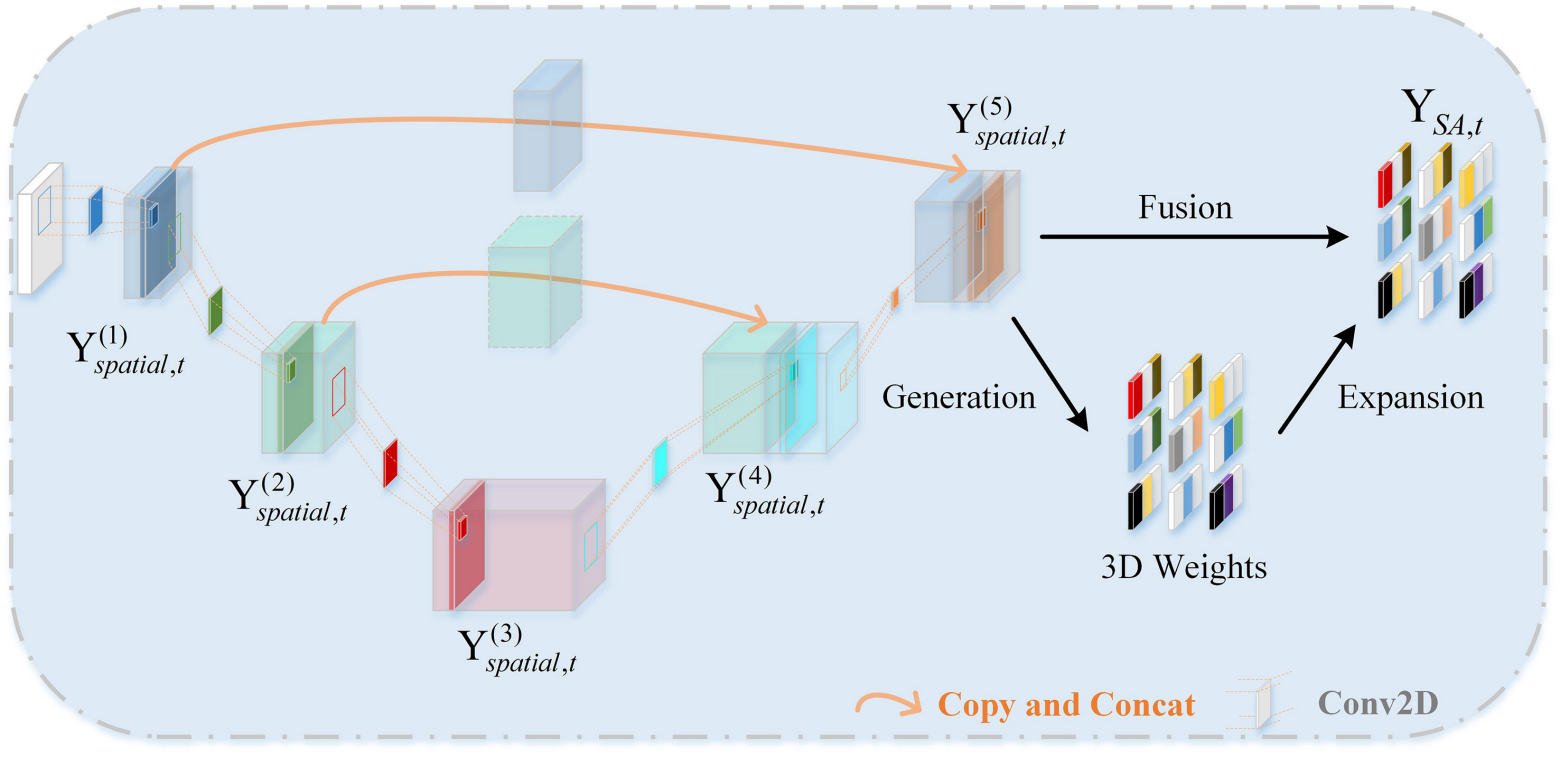
The framework diagram of the SAFE.

The role of the Spatial Attention Feature Extractor (SAFE) is to extract spatial features from the EEG data to adaptively capture the most crucial EEG channels within the 3D patch. As shown in Figure 3. It primarily consists of two parts, namely the U-shaped Continuous Convolution Fusion module (UCCF) and the parameter-free Spatial Attention Module (SA). And we provided detailed explanations for the research motivation behind each module and their specific details.

#### 2.3.1 U-shaped continuous convolution fusion module

Due to the relatively small dimensions (9 × 9) of the 3D features after 3D mapping, applying conventional convolution operations as used in image processing directly would result in an excessively large receptive field. Such approaches have failed to capture enough fine-grained features (Dumoulin and Visin, 2016; O'shea and Nash, 2015). There could be two potential solutions to this problem. The first involves using shallow networks with small kernel sizes, which is unsatisfactory in terms of capturing spatial features (Tan and Le, 2019). The other one is to employ a deeper network, but this could lead to issues such as overfitting and gradient explosion (Fan et al., 2024). For images, adjacent pixels often exhibit minimal differences, so researchers do not need to excessively emphasize fine-grained features when processing images. while the 3D EEG features are distinct, each “pixel” represents an individual channel, and the relationships between channels are subtle and intricate. Despite the secondary processing of channels based on electrode placement, some neighboring electrodes may have weak relationships, while some distant electrodes may have strong connections (Jin et al., 2024). So, we need to focus more on the fine-grained features of the entire 3D patch. Skip connections (Ronneberger et al., 2015) is introduced into the network, as shown in Figure 3. There are two branches, the “channel expansion” branch on the left and the “channel contraction” branch on the right. These two branches are connected via a cascade operation. It is worth noting that, in the corresponding layers of the two branches, there is a fusion of intermediate features, creating skip connections. This approach ensures that the network can capture sufficient fine-grained features while mitigating overfitting to some extent.

In previous sections, we rearranged the DE features to obtain 3D patches. The input data for UCCF is represented as $X_{t}\in {\mathbb{R}}^{\left(n\times B\times h\times w\right)}$, where n is the number of windows, B is the number of frequency bands, h and w are the length and width of the two-dimensional feature matrix, respectively. The three-dimensional structure of the EEG patches integrate spatial features, frequency band features and time features to facilitate network extraction and processing. Specifically, the structure of each patch is *P*_*i*_ = (*x*_1, 1_, *x*_1, 2_, …, *x*_*h, w*_), where $x_{i,j}\in {\mathbb{R}}^{\left(h\times w\right)}$. Let ${\text{Y}}_{\text{spatial,}\text{t}}^{\left(k\right)}$ represent the output of the k-th layer spatial convolution, ${\text{Y}}_{\text{spatial,}\text{t}}^{\left(k\right)}\in {\mathbb{R}}^{\left(n\times B_{k}\times h_{k}\times w_{k}\right)}$, where *B*_*k*_, *h*_*k*_, *w*_*k*_ represent the number, length and width of feature maps obtained as the output of the k-th layer. ${\text{Y}}_{\text{spatial,}\text{t}}^{\left(k\right)}$ can be defined as:


(4) $$\begin{array}{l} {\text{Y}}_{\text{spatial},\text{t}}^{\left(k\right)}={\text{Φ}}_{\text{ReLU}}\left(Conv2D\left({\text{Y}}_{\text{spatial},t}^{\left(\text{k}-1\right)},{\text{S}}^{\left(k\right)}\right)\right) \end{array}$$

Where *S*_*k*_ is the kernel size of the k-th convolutional layer. ${\text{Y}}_{\text{spatial,}\text{t}}^{\left(k-1\right)}$ represents the output of the previous layer. Conv2D(.) represents a two-dimensional convolution operation. Φ_ReIU_(.) is RELU activation function. Due to the existence of skip connections, the spatial convolution output formulas for the 4th and 5th layers are different, defined as follows:


(5) $$\begin{array}{l} {\text{Y}}_{\text{spatial},\text{t}}^{\left(4\right)}={\text{Φ}}_{\text{ReLU}}\left(Conv2D\left(\mathrm{CAT}\left({\text{Y}}_{\text{spatial},t}^{\left(3\right)},{\text{Y}}_{\text{spatial},t}^{\left(2\right)}\right),{\text{S}}^{\left(4\right)}\right)\right) \end{array}$$

(6) $$\begin{array}{l} {\text{Y}}_{\text{spatial},\text{t}}^{\left(5\right)}={\text{Φ}}_{\text{ReLU}}\left(Conv2D\left(\mathrm{CAT}\left({\text{Y}}_{\text{spatial},t}^{\left(4\right)},{\text{Y}}_{\text{spatial},t}^{\left(1\right)}\right),{\text{S}}^{\left(5\right)}\right)\right) \end{array}$$

Where CAT(A, B) represents the concatenation operation, meaning A and B are joined along a certain dimension to form a new vector, which is then used as a new input and passed through the corresponding spatial convolutional layer.

#### 2.3.2 Spatial attention

The EEG signal is essentially a bioelectrical signal, and its generation is related to the neural mechanism of the human brain. When a neuron becomes active, it often leads to a spatial inhibition in the surrounding area to suppress neighboring neurons (Yang et al., 2021). Therefore, neurons that exhibit this inhibitory effect on their surroundings are relatively expected to have higher weights. Our attention module, based on this theory, employs a parameter-free method to directly compute the attention weights for 3D inputs. The specific approach is as follows:

To estimate the importance of each individual neuron, based on the spatial inhibition phenomenon, a straightforward approach is to measure the linear separability between the target neuron and other neurons. Based on linear separability theory, let *e*_*t*_ denote the energy of neuron t, establish the energy equation as follows:


(7) $$\begin{array}{l} e_{t}\left(w_{t},b_{t},\text{y},x_{i}\right)={\left(y_{t}-\hat{t}\right)}^{2}+\frac{1}{M-1}\overset{M-1}{\underset{i=1}{\sum }}{\left(y_{o}-{\hat{x}}_{i}\right)}^{2} \end{array}$$

where $\hat{t}=w_{t}t+b_{t}$ and ${\hat{x}}_{i}=w_{t}x_{i}+b_{t}$ are the linear transformations of *x*_*i*_ and t, respectively. And t is the target neuron for which the degree of correlation needs to be calculated and *x*_*i*_ represents other neurons except t, i represents the neuron index, *w*_*t*_ and *b*_*t*_ are the weight coefficients and bias coefficients in linear transformation. M represents the total number of channels in a specific frequency band. When $\hat{t}$ equals *y*_*t*_ and $\hat{x_{i}}$ equals *y*_0_, this formula can obtain the minimum value, which is equivalent to finding the linear separability between the target neuron $\hat{t}$ and other neurons $\hat{x_{i}}$. In order to facilitate calculation, the two constants *y*_*o*_ and *y*_*t*_ are set to −1 and 1, respectively. 0 can be rewritten as


(8) $$\begin{array}{l} e_{t}\left(w_{t},b_{t},\text{y},x_{i}\right)=\frac{1}{M-1}\sum_{i=1}^{M-1}{\left(-1-\left(w_{t}x_{i}+b_{t}\right)\right)}^{2}\\ \;+{\left(1-\left(w_{t}t+b_{t}\right)\right)}^{2}+\lambda w_{t}^{2} \end{array}$$

where $\lambda w_{t}^{2}$ is the regularization term, λ is a constant. We can quickly get the analytical solution as:


(9) $$\begin{array}{l} w_{t}=-\frac{2\left(t-u_{t}\right)}{{\left(t-u_{t}\right)}^{2}+2\sigma_{t}^{2}+2\lambda } \end{array}$$

(10) $$\begin{array}{l} b_{t}=-\frac{1}{2}\left(t+u_{t}\right)w_{t} \end{array}$$

Among them, $u_{t}=\frac{1}{M-1}\overset{M-1}{\underset{i=1}{\sum }}x_{i}$, $\sigma_{t}^{2}=\frac{1}{M-1}\overset{M-1}{\underset{i=1}{\sum }}{\left(x_{i}-u_{t}\right)}^{2}$. This is also the condition under which the energy function e attains its minimum value. So, the minimum energy can be obtained as:


(11) $$\begin{array}{l} e_{t}^{*}=\frac{4\left({\hat{\sigma }}^{2}+\lambda \right)}{{\left(t-u_{t}\right)}^{2}+2\sigma_{t}^{2}+2\lambda } \end{array}$$

Finally, based on this minimum energy, the importance of each neuron can be quantified, expressed as 1/e*. In order to further adapt the attention mechanism to the attention regulation method in the mammalian brain, gain and scaling are used to enhance features, as shown in Equation 12.


(12) $$\begin{array}{l} \tilde{\text{X}}=\mathrm{sigmoid}\left(\frac{1}{\text{E}}\right)⊙\text{X} \end{array}$$

Where X is the original input feature map, and $\tilde{X}$ represents the refined feature map after applying the attention module. E represents all neuron nodes, i.e., all EEG channels. sigmoid() The sigmoid function is added to map the values in E to the range of 0–1. Therefore, the spatial attention module can be expressed as:


(13) $$\begin{array}{l} Y_{SA,t}=MP\left(SA\left({\text{Y}}_{\text{spatial},\text{t}}\right)\right) \end{array}$$

where SA(.) represents the spatial attention mechanism module. MP(.) represents max pooling operation. We then perform a flattening operation on the resulting output.

### 2.4 Temporal attention feature extractor

As shown in Figure 1, the temporal attention feature extractor consists of position encoding embeddings and several identical temporal encoding layers. In each temporal encoding layer, contains multiple self-attention layers, Feedforward Neural Network (FFN) layers, and LayerNorm layers. Additionally, residual connections are applied throughout the network.

After SAFE, the vector already contains spatial feature information. Each vector *Y*_*t*_ is treated as an individual token, and these T tokens are combined to form the input sequence for TAFE. In order to capture temporal feature information across the entire sequence, similar to the BERT structure (Devlin et al., 2018), a learnable class token is added at the very beginning of the input sequence to obtain aggregated information from the entire input, i.e.,


(14) $$\begin{array}{l} {\text{Z}}_{c}=\left[{\text{Y}}_{\text{class}},{\text{Y}}_{1},{\text{Y}}_{2},\ldots ,{\text{Y}}_{t},\ldots ,{\text{Y}}_{T}\right] \end{array}$$

where *Y*_class_ is class token, *Y*_*t*_ is the output of SAFE, and *Z*_*c*_ represents the sequence after adding the class token. $Y_{\text{class}}\in {\mathbb{R}}^{\left(n\times 1\times d\right)}$, $Y_{t}\in {\mathbb{R}}^{\left(n\times 1\times d\right)}$, where d is the dimension of the embedding vector. The self-attention mechanism, by itself, only considers the relationships between tokens in the input sequence but does not take into account their specific positions. Positional encoding is added to the TAFE to address the issue of the self-attention mechanism not capturing positional information within the input sequence. That is, the sequence *Z*_*p*_ after adding position coding is


(15) $$\begin{array}{l} {\text{Z}}_{p}={\text{Z}}_{c}+\text{P} \end{array}$$

where *P* ∈ ℝ^(*n*×(*T*+1) × *d*)^ is the position encoding. The multi-head self-attention mechanism (Wang et al., 2023) transforms the same input matrix Z into Q, K, and V matrices to calculate the corresponding attention weights for each element in the sequence. The calculation of the output result for the h-th head's attention weights can be defined as follows:


(16) $$\begin{array}{l} A_{h}=\mathrm{Attention}\left(Q_{h},K_{h},V_{h}\right)=\mathrm{Softmax}\left(\frac{Q_{h}{K_{h}}^{T}}{\sqrt{d_{k}}}\right)V_{h} \end{array}$$

Where h=1,2,...,H. $\sqrt{d_{k}}$ represents the scaling coefficient of the elements in $Q_{h}{K_{h}}^{T}$. It changes the variance of all elements to 1 so that the gradient value remains stable during the training process. Q, K, and V are obtained by linear transformation from the input Z. We can obtain it through Equation 17:


(17) $$\begin{array}{l} \left\{ \begin{array}{c} Q_{h}=\mathrm{Linear}\left(Z\right)\\ K_{h}=\mathrm{Linear}\left(Z\right)\\ V_{h}=\mathrm{Linear}\left(Z\right) \end{array}\right. \end{array}$$

The outputs are concatenated and then passed through a fully connected layer, i.e.,


(18) $$\begin{array}{l} \mathrm{MSA}\left(\text{Q},\text{K},\text{V}\right)=\text{FC}\left(\text{CAT}\left(\text{A}1,\text{A}2,\ldots ,{\text{A}}_{\text{H}}\right)\right) \end{array}$$

Therefore, the output *Z*_*A*_ of MSA and the output *Z*_*F*_ of FFN can be defined as:


(19) $$\begin{array}{l} Z_{\text{A}}=\text{LN}\left(\text{MSA}\left(\text{Z}\right)+\text{Z}\right) \end{array}$$

(20) $$\begin{array}{l} Z_{\text{F}}=\text{LN}\left(\text{Linear}\left({\text{Φ}}_{\text{GELU}}\left(\mathrm{Linear}\left(\text{F}\right)\right)\right)+\text{F}\right) \end{array}$$

LN represents the LayerNorm. After iterating H temporal encoding layers, take out the class token for classification.

Finally, we conduct a qualitative analysis of the computational complexity of the entire model. Since the space complexity and time complexity of pooling layer and linear layer is much smaller than that of CNN and TAFE, the computational complexity of HASTF is


(21) $$\begin{array}{l} O_{\text{Time}}=\sum O_{CNN}+O_{\text{TAFE}}=\sum O\left(H_{\text{out}}^{2}•k^{2}•C_{\text{in}}•C_{\text{out}}\right)\\ \;+O\left(T^{2}d+Td^{2}\right) \end{array}$$

(22) $$\begin{array}{l} O_{\text{Space}}=\sum O_{CNN}+O_{\text{TAFE}}=\sum O\left(k^{2}•C_{\text{in}}•C_{\text{out}}\right)+O\left(h•d^{2}\right) \end{array}$$

where *H*_*out*_ is the output feature map size, *k* is the filter size, *C*_*in*_ and *C*_*out*_ are the number of input and output channels, respectively. T and d are the input sequence length and dimension size of TAFE.

## 3 Experiments

### 3.1 Datasets

In order to rigorously assess the performance of the proposed algorithm, we have selected two public datasets widely used within the realm of EEG-based emotion recognition research. The details of these two datasets will be provided in the subsequent section.

#### 3.1.1 DEAP dataset

DEAP dataset (Koelstra et al., 2011) comprises 32 participants (16 males and 16 females) with an average age of 26.9. It uses music videos as the primary stimulus method. Each participant underwent 40 trials, with each trial lasting for 63 s. The initial 3 s are the time to obtain baseline data, and the last 60 s are the time for music stimulation. After each trial, each participant was asked to rate their emotional state in this trial on four dimensions (arousal, valence, dominance, and liking) from 1 to 9. The device has 40 channels, the first 32 channels are used to collect EEG signals with a sampling frequency of 512 Hz. In this study, we chose 5 as the threshold, dividing the labels into two binary classification problem, that is, HA/LA and HV/LV.

#### 3.1.2 SEED dataset

The SEED Emotion Dataset (Zheng and Lu, 2015) is provided by SJTU. It elicits three types of emotions—positive, neutral, and negative—through clips of movie segments, using videos as the primary stimulus. The dataset includes 15 participants (seven males and eight females) with an average age of 23.27. Each participant undergoes one session per week, for a total of three sessions. Each session involves watching 15 movie clips, each approximately 4 min long, a duration considered sufficient to induce and maintain the participants' emotional responses without causing fatigue or emotional decay. The process for each movie clip includes a 5-s prompt before the start, 4 min of the movie clip, 45 s of self-assessment, and 15 s of rest. The data is gathered using 62 channels device at a sampling frequency of 1,000 Hz.

### 3.2 Data preprocessing

For SEED. The author has manually removed EMG and EOG artifacts from the raw data. Subsequently, we down-sampled the data to 200 Hz and employed a band-pass filter to eliminate high-frequency noise. When adding labels, positive, neutral, and negative emotions are represented by 1, 0, and −1, respectively. For DEAP. In the preprocessing stage, Electrooculogram (EOG) artifacts are removed, and then is down-sampled to 128 Hz, so each trial has 63 × 128 = 8,064 sampling points. The data dimensions of each subject are 40 × 40 × 8,064. Finally, for the subjects' ratings from 1 to 9, we chose 5 as the threshold, categorizing the labels into high/low arousal and high/low valence, thereby transforming it into a binary classification problem.

### 3.3 Evaluation metrics

We use two of the most commonly used evaluation metrics to evaluate and compare models. Accuracy represents the ratio between the number of samples correctly classified by the model and the total number of samples. Standard deviation (Std) is a statistical metric used to measure the degree of data dispersion within a dataset, indicating the degree of deviation from the mean value. The calculation formulas for both are as follows:


(23) $$\begin{array}{l} \text{Accuracy}=\frac{TP+TN}{TP+FP+TN+FN} \end{array}$$

where TP and TN are true positive and true negative, respectively, FP and FN are false positive and false negativem, respectively.


(24) $$\begin{array}{l} \text{Std}=\sqrt{\frac{\sum_{i=1}^{N}{\left(x_{i}-\mu \right)}^{2}}{N}} \end{array}$$

Where *X*_*i*_ represents the i-th data. μ represents the average of all data. N represents the total number of all data points.

In the ablation experiment, we also used one-way ANOVA to detect whether there are significant differences among the various modules. One-way ANOVA is a statistical analysis method that compares whether there are significant differences in the means between two or more groups by calculating within-group variance and between-group variance.

### 3.4 Implementation details

We employ 5-fold cross-validation approach in all experiments. For the DEAP dataset, each subject participated in 40 trials, while in the SEED dataset, each subject took part in 15 trials. All trial data are shuffled and divided into five subsets. Each subset take turns serving as the test set, while the remaining four subsets are used as the training data, ensuring against randomness and data leakage. The final result is calculated as the average of the results from these 5-folds.

For SAFE. UCCF consists of five convolutional layers. The kernel size of the first four layers is all set to (3,3), stride = (1,1), padding = (1,1). We change the kernel size of the last one to (1,1), with no other variations in comparison to the preceding layers. Skip connections are applied between the outputs of the first layer and the fourth layer, as well as between the outputs of the second layer and the third layer. Throughout the entire convolution process, the spatial dimensions remain invariant.

For TAFE. Random numbers conforming to a standard normal distribution within the range of 0–1 are generated to initialize the class token and the position embedding. There are a total of six temporal encoding layers. And the number of heads in the MSA is set to 8. The dimensions of the QKV matrices and that of the linear layers within the FFN are specified as 256 and 128, respectively. We also utilize LayerNorm and the GELU activation function.

During the experiment, we use the cross-entropy loss function and AdamW optimizer. The initial learning rate was set to 0.0001, with a weight decay of 0.0001. The batch size is 32 and the number of epoches is 100. We trained and tested the proposed model on NVIDA TESLA T4 Tensor Core GPU, and Pycharm, Python 3.8, Pytorch 1.13.0 were used for algorithm implementation.

## 4 Results analysis

In this section, we first report the performance of the proposed method on the DEAP dataset and SEED dataset, and compare it with the current state-of-the-art methods. Additionally, to investigate the roles of different modules in HASTF, we then conduct ablation experiments on each proposed module and use statistical methods to further elucidate the functions of these individual modules. Finally, we perform interpretability analysis on the entire network, display and analyze the activation states of brain regions and crucial channals corresponding to different emotional states and different subjects.

### 4.1 Experimental results on DEAP and SEED

**Figure 4:**
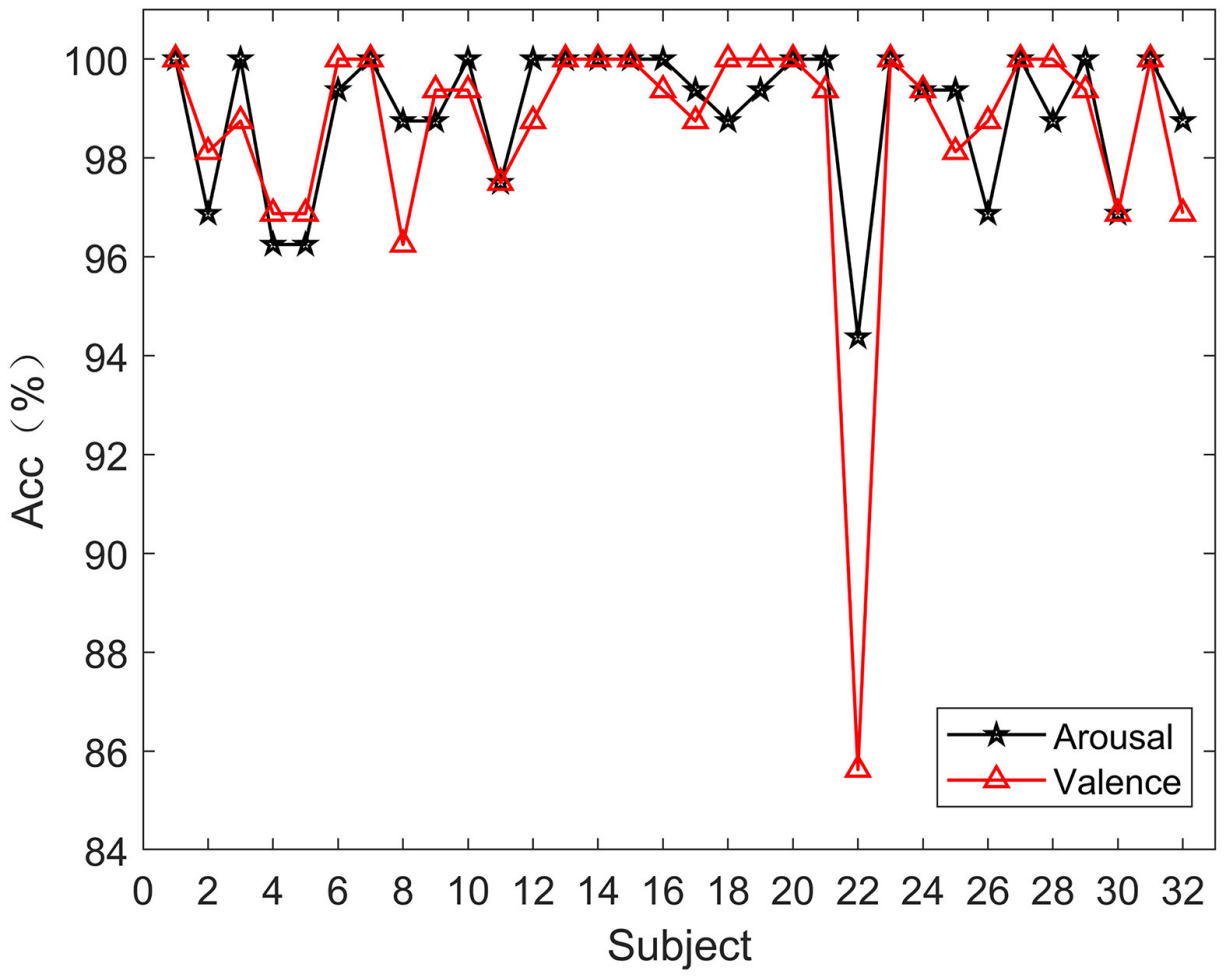
Experimental results on the DEAP dataset.

**Figure 5:**
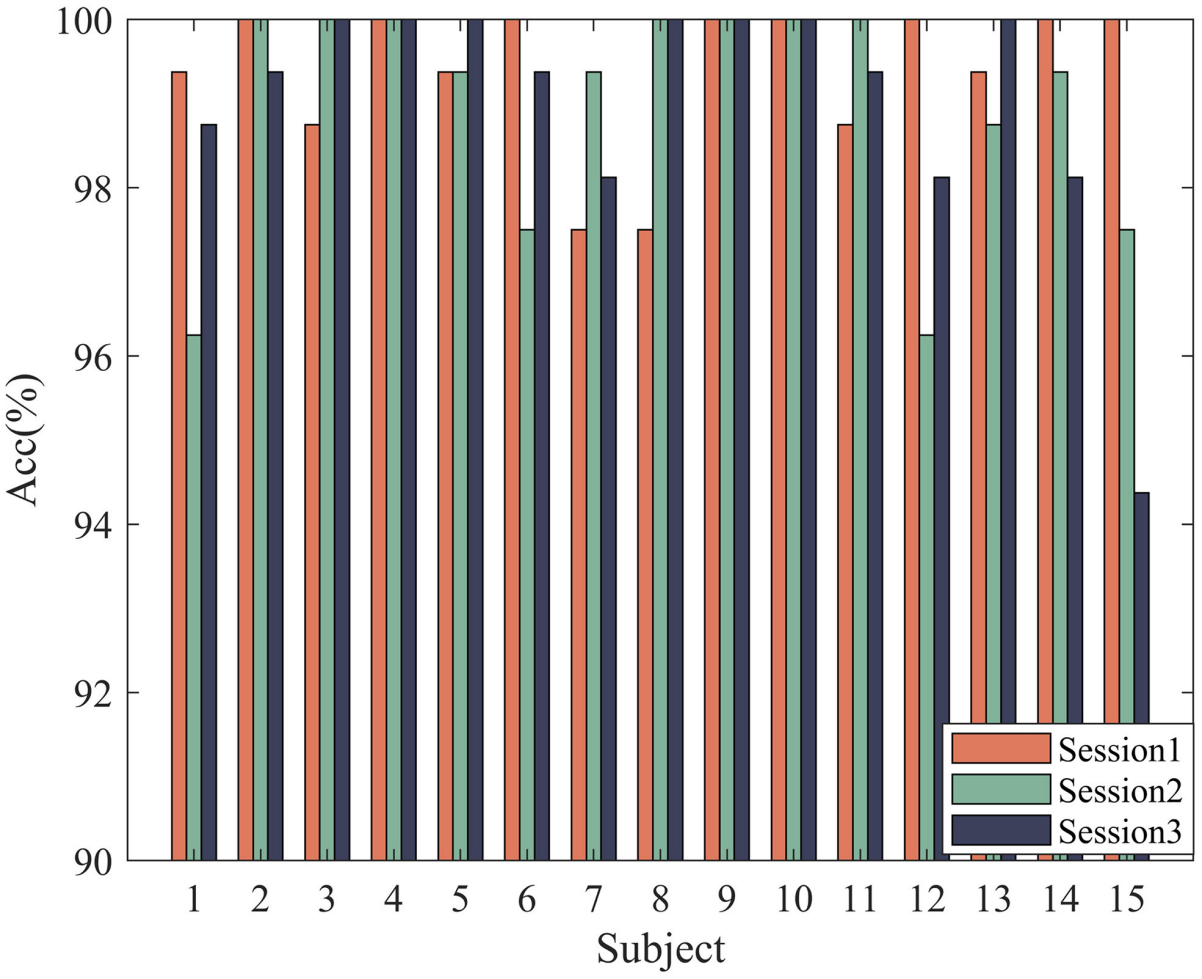
Experimental results on the SEED dataset.

In order to verify the effectiveness of the proposed method, according to the experimental method introduced in Section 3, 5-fold cross-validation is used to evaluate our model. The experimental results of 32 subjects in the DEAP dataset are shown in Figure 4. Based on all the results, HASTF achieves an impressive average accuracy of 98.93% ± 1.45% on the Arousal dimension and 98.57% ± 2.60% on the Valence dimension. From the graph, it can be observed that subjects 1, 6, 7, 13, 14, 15, 18, 19, 20, 23, 27, 28, and 31 achieve a 100% accuracy in Valence. Additionally, half of the subjects achieve a 100% accuracy in Arousal, and for most subjects, the accuracy is above 96% in both labels. It's worth noting that the 22nd subject has a low recognition accuracy in both labels. However, we have observed similar results in previous studies (Yang et al., 2018a; Shen et al., 2020; Tao et al., 2020; Zhong et al., 2023), where the 22nd subject's recognition accuracy is also subpar. This may be attributed to the subject's inaccurate self-assessment of their emotional state after the experiment. We then verify HASTF on the SEED dataset, as shown in Figure 5, which displays the recognition accuracy for 15 subjects across three sessions. From the bar chart, it is evident that the results across 45 sessions are quite stable, with over 90% of sessions achieving recognition accuracy above 98%. This demonstrates that HASTF performs well for all sessions in the SEED dataset, resulting in an average recognition accuracy of (99.12% ± 1.25%).

### 4.2 Method comparison

In this section, we aim to further demonstrate the superiority of our method by conducting a comparative analysis with the most representative emotion recognition methods. This comparison encompasses widely used traditional machine learning methods Support Vector Machines (SVM) as well as various deep learning methods proposed by researchers in recent years. In the following content, we provide a brief introduction to these methods.

a. Para-CNN-LSTM (Yang et al., 2018b): It proposed a parallel network of CNN and LSTM and inputs the extracted DE features into the network.

b. 4D-CRNN (Shen et al., 2020): It used a CNN and LSTM sequential structure, comprising five CNN layers and two LSTM layers, to extract DE features as the input to the network.

c. ATDD-LSTM (Du et al., 2020): It added an attention mechanism module to LSTM, proposed an attention-based LSTM and domain discriminator, and also inputted DE features into the proposed network.

d. ST-GCLSTM (Feng et al., 2022): It utilized the biological topological information between brain regions, extracted spatial features using GCN from multiple EEG channels, and employed bidirectional LSTM with attention mechanisms to extract temporal features.

e. 4D-ANN (Xiao et al., 2022): It incorporated spatial attention mechanisms into the 4D-CNN-LSTM sequential structure, adapting weights for different brain regions and frequency bands.

f. MTCA-CapsNet (Li C. et al., 2022): It used a capsule network as the base model and incorporated attention mechanisms to extract feature probability maps from different channels. A multi-task learning network based on capsule network and attention mechanism was proposed, which can effectively learn the intrinsic relationship between different tasks of EEG signals.

g. FPN (Hou et al., 2022): It extracted DE features as fundamental features, then constructed a feature matrix to obtain inter-channel correlations, and builded FPN network to obtain distinctive EEG features for emotion classification.

h. ACRNN (Tao et al., 2020): It comprehensively considered the spatial information, temporal information, and attention mechanism of EEG signals, integrated the attention mechanism into CNN, and combined it with RNN for emotion recognition.

i. STFCGAT (Li et al., 2023): It integrated multi-head attention mechanism into the graph convolutional neural network, and considered multi-band DE features and FC features to extract powerful graph structure information.

j. Bi-AAN (Zhong et al., 2023): It established a bihemispheric asymmetric attention network based on the asymmetry of the brain and combined with the Transformer architecture. This simulates the attention differences between brain hemispheres, providing a more precise description of distinctive emotional representations.

k. CSGNN (Lin et al., 2023): It combined the advantages of 1D convolution and graph convolution, proposed an improved dynamic channel selection graph convolutional network. This approach reduces the computational cost of emotion recognition while maintaining high recognition accuracy.

l. MFBPST-3D-DRLF (Miao et al., 2023): It proposed a novel multi-band parallel spatio-temporal 3D deep residual learning framework to extract high-level abstract features and achieve accurate classification.

m. TDMNN (Ju et al., 2024): Ju et al. incorporated the slow changes of emotions over time as a priori knowledge into emotion recognition. They enhanced the model's ability to capture subtle changes in EEG features over time through MMD evaluation of temporal differences and a multi-branch strategy.

n. FTSCN (Yang et al., 2024): It combines factorization machine and separable convolution into TCN and develops a Factorization Temporal Separable Convolution Network. It can not only capture long-term dependencies in time series data, but also increase the model's expressive power in feature dimensions.

o. PGCN (Jin et al., 2024): This paper proposes a pyramid structure model based on GCN, it combines the 3D topological relationship of the brain to aggregate features at the local, central and global levels.

p. SST-Emo (Peng et al., 2024): SST-Emo leverages spectrum-based spatial channel attention and time continuity encoding mechanisms to fully utilize the spectral, spatial, and temporal features of EEG signals, demonstrating excellent performance in emotion analysis tasks.

The performance of each algorithm on SEED dataset and DEAP dataset are shown in Tables 1, 2, respectively. The comparison results clearly indicate that our approach outperforms other methods. Overall, compared to traditional machine learning method, our approach shows a significant performance improvement, surpassing the SVM method by approximately 14%. In comparison to other deep learning methods, our approach not only exhibits higher accuracy and lower standard deviation but also consistently performs well on both the DEAP and SEED datasets. In more detail, first, HASTF outperforms all CNN-based methods. Compared to the Para-CNN-LSTM model, HASTF achieves an average accuracy improvement of 8.18% and 6.31% for DEAP-Arousal and DEAP-Valence, respectively. The corresponding improvements of 4D-CRNN are 4.35% and 3.12% for the DEAP dataset and 4% for the SEED dataset. It also surpasses TDMNN by 0.59% and 1.92% on the DEAP and SEED datasets, respectively. Second, HASTF also surpasses all GCN-based methods. On the SEED dataset, it exceeds DGCNN, CSGCN and PGCN by 8.72%, 8.90%, and 2.19%, respectively, showing significant superiority. These enhancements are primarily attributed to the superior capacity of our model in capturing the overall contextual information within EEG signals.

**Table 1:** The performance of each algorithm on the DEAP dataset.

**Methods**	**Year**	**DEAP-arousal**		**DEAP-valence**	
				**Acc (%)**	**Std (%)**	**Acc (%)**	**Std (%)**
SVM	2019	86.75	–	84.05	–
Para-CNN-LSTM	2018	90.8	3.08	91.03	2.99
4D-CRNN	2020	94.58	3.69	94.22	2.61
ATDD-LSTM	2022	90.87	11.32	90.91	12.95
4D-ANN	2022	97.39	1.75	96.90	1.65
MTCA-CapsNet	2022	97.41	1.47	97.24	1.58
FPN	2022	96.97	–	94.29	–
ST-GCLSTM	2023	95.04	–	95.52	–
ACRNN	2023	93.38	3.73	93.72	3.21
STFCGAT	2023	95.04	3.02	95.70	3.36
Bi-ANN	2023	96.96	**1.29**	96.63	**1.30**
FTSCN	2024	97.39	1.93	97.55	1.65
TDMNN	2024	98.25	2.85	98.08	2.13
SST-emo	2024	96.28	2.34	95.25	2.93
Ours	–	**98.93**	1.45	**98.57**	2.60

The bold font in the table represents the best in the results.

**Table 2:** The perform of each algorithm on the SEED dataset.

**Methods**	**Year**	**SEED**	
				**Acc (%)**	**Std (%)**
4D-CRNN	2020	94.58	6.16
DGCNN	2020	90.40	8.49
ATDD-LSTM	2022	91.08	6.43
4D-ANN	2022	96.25	1.86
FPN	2022	97.12	–
ST-GCLSTM	2023	96.72	–
CSGNN	2023	90.22	3.67
MFBPST-3D-DRLF	2023	96.67	2.80
STFCGAT	2023	99.11	**0.83**
FTSCN	2024	89.13	4.49
TDMNN	2024	97.20	1.57
PGCN	2024	96.93	5.11
Ours	–	**99.12**	1.25

The bold font in the table represents the best in the results.

Comparing our method to STFCGAT, Bi-AAN and SST-emo, all four employ multi-head attention mechanisms to extract temporal information. HASTF outperforms STFCGAT and SST-emo by almost 3% on the DEAP, and it surpasses Bi-AAN by 1.97% and 0.71% in arousal and valence, respectively. This suggests that the proposed UCCF with spatial attention and skip connections is better at extracting deep and fine-grained emotional features. It's worth noting that STFCGAT exhibits similar accuracy to our method on the SEED dataset and has a lower standard deviation by 0.42%, but our method outperforms STFCGAT by 2% for two dimensions on the DEAP dataset. This further demonstrates that our method has high generalization performance and is suitable for emotion recognition generated by different stimuli.

Comparing our method with 4D-ANN, ACRNN, ST-GCLSTM, MFBPST-3D-DRLF, MTCA-CapsNet, these methods all use attention mechanisms to obtain the information of EEG channels, but our method outperforms these models by 0.1%–5.55% on the DEAP dataset and exhibits a superior improvement of over 2% on the SEED dataset, with higher recognition accuracy. This can also prove from the side that the parameter-free attention mechanism we use can help the network acquire key channels more precisely, Additionally, this also indicates that HASTF provides more comprehensive extraction of crucial information in the time domain.

### 4.3 Ablation experiments

**Figure 6:**
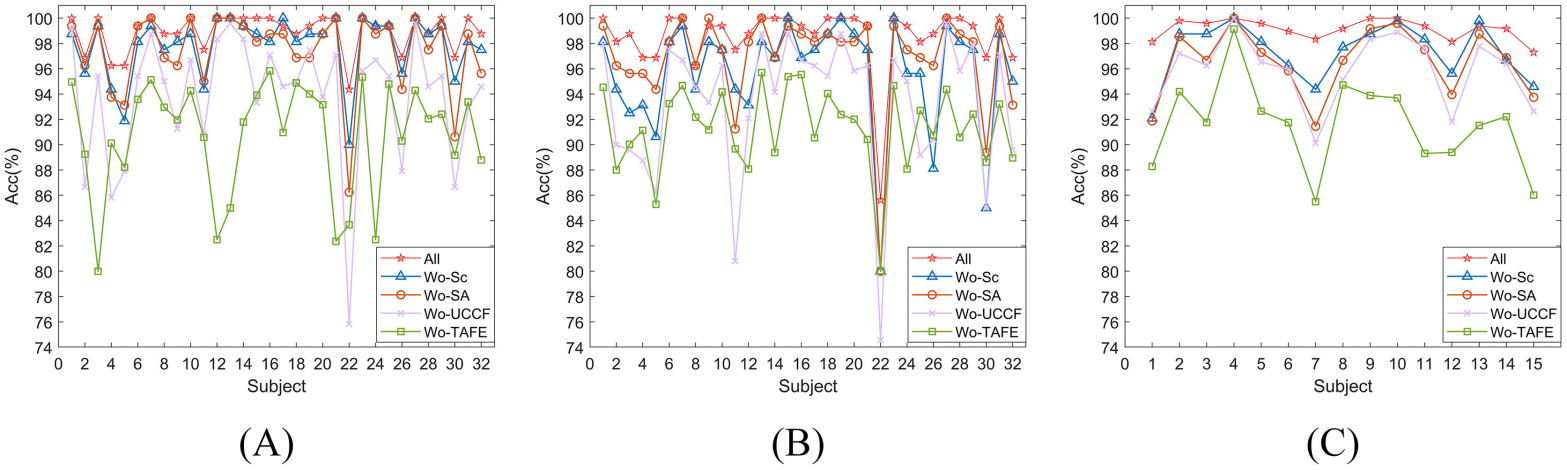
The results of the ablation experiments on **(A)** DEAP-arousal **(B)** DEAP-valence **(C)** SEED.

In this section, we conducted various ablation experiments to analyze in more detail the impact of different modules in the network on the results, further exploring how our network achieves such performance. In Section 2, our proposed method is divided into two main parts: SAFE and TAFE, with SAFE consisting of UCCF and Spatial Attention (SA). Therefore, we conducted ablation experiments on the Skip connection, SA, UCCF, and SAFE modules, which are All: HASTF, Wo-Sc: Without Skip connection, Wo-SA: Without-SA, Wo-UCCF: Without-UCCF, Wo-TAFE: Without TAFE. Table 3 gives the overall experimental design. The results corresponding to the four test plans are shown in Figure 6. Let's first focus on the role of skip connections and spatial attention in UCCF. It can be calculated from the experimental results that when UCCF does not have skip connections, the average accuracy of the model on the DEAP dataset decreases by 2.00%, and the accuracy on the SEED dataset decreases by 1.81%. This is due to the fact that when the fusion of shallow and deep network features is canceled, with the increase in the number of iterations, some spatial information is lost, leading to a decrease in accuracy. When UCCF lacks the spatial attention, the average accuracy on the DEAP dataset decreases by 1.62%, and the accuracy on the SEED dataset decreases by 2.59%. This clearly highlights the importance of the attention mechanism. The existence of the attention mechanism allows the network to follow the contribution of each channel gives each channel the weight it deserves, adaptively selecting crucial channels for emotion recognition instead of treating all channels equally. Then, we examine the role of UCCF and TAFE. Without UCCF, i.e., only the temporal domain features in the EEG data are extracted, the accuracy of the model dropped by 5.19% and 3.3%, respectively, on DEAP and SEED. Without SAFE, which means extracting only the spatial domain features from EEG data, the model's accuracy drops by 7.75% on DEAP and 7.52% on SEED. Obviously, the lack of TAFE has a greater impact on the model. This may be because EEG data is inherently collected over time, making temporal information more accessible and capable of effectively representing the deeper emotional features.

**Table 3:** Design of ablation experiments.

	**Skip-connection**	**SA**	**UCCF**	**SAFE**
Wo-Sc	×	✓	✓	✓
Wo-SA	✓	×	✓	✓
Wo-UCCF	–	–	×	✓
Wo-SAFE	✓	✓	✓	×
All	✓	✓	✓	✓

To better understand the impact of each module on the final results, we use one-way ANOVA to study the significant differences after each module is removed at the 95% confidence level. We set the null hypothesis (H0) as the means of each group being equal, that is, removing different modules has no significant impact on model performance. The corresponding alternative hypothesis (H1) is that at least one group's mean is significantly different from the others, that is, removing certain modules significantly affects model performance. The statistical results are shown in Table 4. For the DEAP dataset, the degrees of freedom within groups (DFW) and between groups (DFB) are 4 and 155, respectively, while for the SEED dataset, DFW and DFB are 4 and 220, respectively. it can be observed that the *p*-values for all three are significantly < 0.05. This indicates that there are statistically significant differences under different module. The high *F*-scores (29.59, 15.67, and 33.37) indicate that the variability between the groups (different models) is much greater than the variability within each group. The corresponding low *p*-values (2.78E-18, 8.70E-11, 9.48E-22) suggest that these differences are statistically significant. Thus, we can reject the null hypothesis and conclude that the removal of different modules leads to significantly different performance outcomes.

**Table 4:** The results of one-way ANOVA.

**Dataset**		**Sum of squares**	**Degree of freedom**	**Mean squares**	***F*** **-score**	***p*** **-value**
DEAP-arousal	Between	1,423.19	4	355.80	29.59	2.78E-18
	With	1,863.92	155	12.03		
	Total	3,287.11	159	–		
DEAP-valence	Between	1,079.51	4	269.88	15.67	8.70E-11
	With	2,670.07	155	17.23		
	Total	3,749.57	159	–		
SEED	Between	1,399.48	4	349.87	33.37	9.48E-22
	With	2,306.81	220	10.49		
	Total	3,706.30	224	–		

To further understand the degree of dissimilarity between different frameworks. we conducted *post-hoc* multiple comparisons using the Games-Howell method after one-way ANOVA. This approach, in combination with the correction for confidence interval biases, provided a more accurate estimation of the confidence intervals for the population parameters. Table 5 displays the results of *post-hoc* multiple comparisons for the two datasets, showing mean differences, standard errors, biases, and other relevant information. This provides a more precise assessment of the contributions of each module to the overall performance. From the results, when a particular module is removed, the average performance decreases compared to the original model. Furthermore, the most significant decrease in performance occurs when the TAFE is omitted.

**Table 5:** The results of multiple comparisons.

**Dataset**			**Mean difference**	**Deviation**	**Standard error**	**Bca95% Confidence interval**	
						**Lower limit**	**Upper limit**
DEAP-arousal	All	Wo-Sc	1.074219	−0.013460	0.522036	0.131240	2.095704
		Wo-Attn	1.503906	−0.035406	0.616266	0.516662	2.610400
		Wo-UCCF	4.459656	−0.033330	0.878871	2.949839	5.994727
		Wo-TAFE	8.235656	−0.039103	0.842052	6.736735	9.715444
DEAP-valence	All	Wo-Sc	2.910156	−0.012861	0.933834	0.992163	4.700305
		Wo-Attn	1.738281	−0.015684	0.825217	0.203462	3.379859
		Wo-UCCF	5.279969	−0.034749	1.136944	3.317446	7.323220
		Wo-TAFE	7.356687	0.001739	0.759507	5.818458	8.777459
SEED	All	Wo-Sc	1.819444	0.016495	0.476962	0.887990	2.843215
		Wo-Attn	2.597222	0.000430	0.535290	1.614596	3.579459
		Wo-UCCF	3.300978	0.034174	0.585036	2.135436	4.558495
		Wo-TAFE	7.523800	−0.010304	0.676748	6.202649	8.855647

### 4.4 Interpretability analysis

In SAFE, we incorporated spatial attention modules, and through the ablation experiments in Section 4.3, we observed that these attention modules significantly improved the network's recognition performance and generalization ability. This suggests that HASTF's high performance is closely related to the adaptive selection of crucial channels through attention mechanisms. In this section, we conduct an in-depth discussion on the added spatial attention mechanism module, explore how it plays a role in emotion recognition, and visually display the results. Additionally, we conducted an analysis of the activation levels in different brain regions corresponding to various emotions. This analysis aims to explore the neural patterns in the human brain associated with emotions and decode the imprints of emotions within the brain regions.

**Figure 7:**
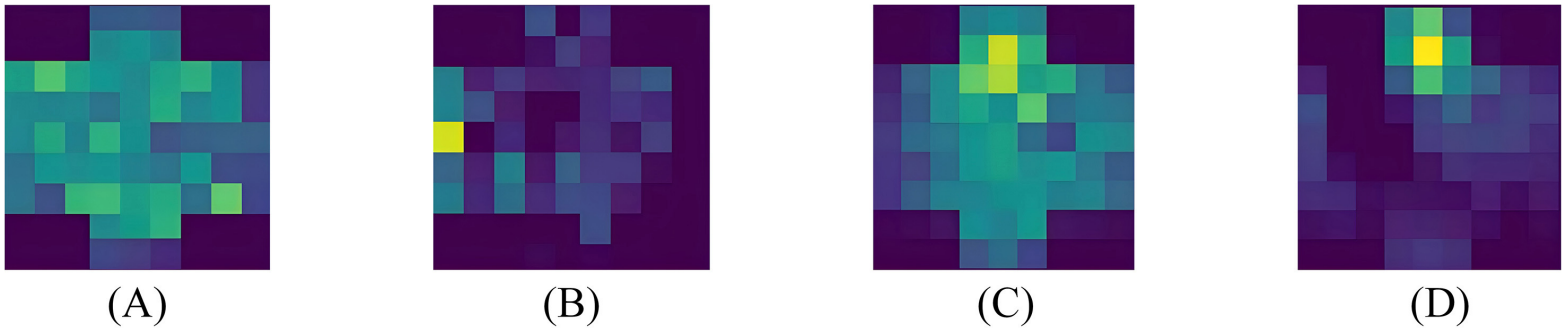
Visualization results on DEAP. **(A)** Pos-Wo SA, **(B)** Pos-SA, **(C)** Neg-Wo SA, **(D)** Neg-SA.

**Figure 8:**
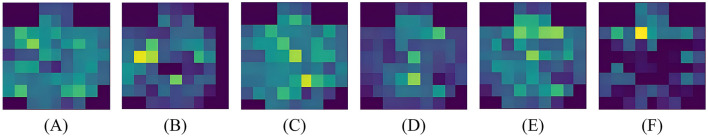
Visualization results on SEED. **(A)** Pos-Wo SA, **(B)** Pos-SA, **(C)** Neu-Wo SA, **(D)** Neu-SA, **(E)** Neg-Wo SA, **(F)** Neg-SA.

Using feature map visualization method. We compared the results when the attention mechanism module is added and when it is not added. The results of adaptive crucial channels and crucial brain regions selection for neutral, negative and positive emotions on two datasets are, respectively, shown in Figures 7, 8.

From the visualized results, it is evident that whether it's the DEAP dataset or the SEED dataset, whether it's positive or negative emotion, when spatial domain attention is not involved, the network's focus is not concentrated on specific channels. There is no clear spatial attention, and the spatial domain contains more noise, as shown in Figures 7A, C, 8A, C, E. When spatial attention is added, the network begins to pay more attention to a certain brain areas or even a certain electrode channel, and the attention to channels in some brain regions weakens, resulting in a clear contrast between bright and dark. The brighter the color, the higher the attention of the network, that is, the higher the activation level of the area; the darker the color, the lower the attention of the network, that is, the lower the activation level of the area; as shown in Figures 7B, D, 8B, D, F. It's worth noting that this selective attention mechanism is similar to the human brain's attention mechanism, which intuitively illustrates the effect and operation of spatial attention.

### 4.5 Emotional neural patterns

**Figure 9:**
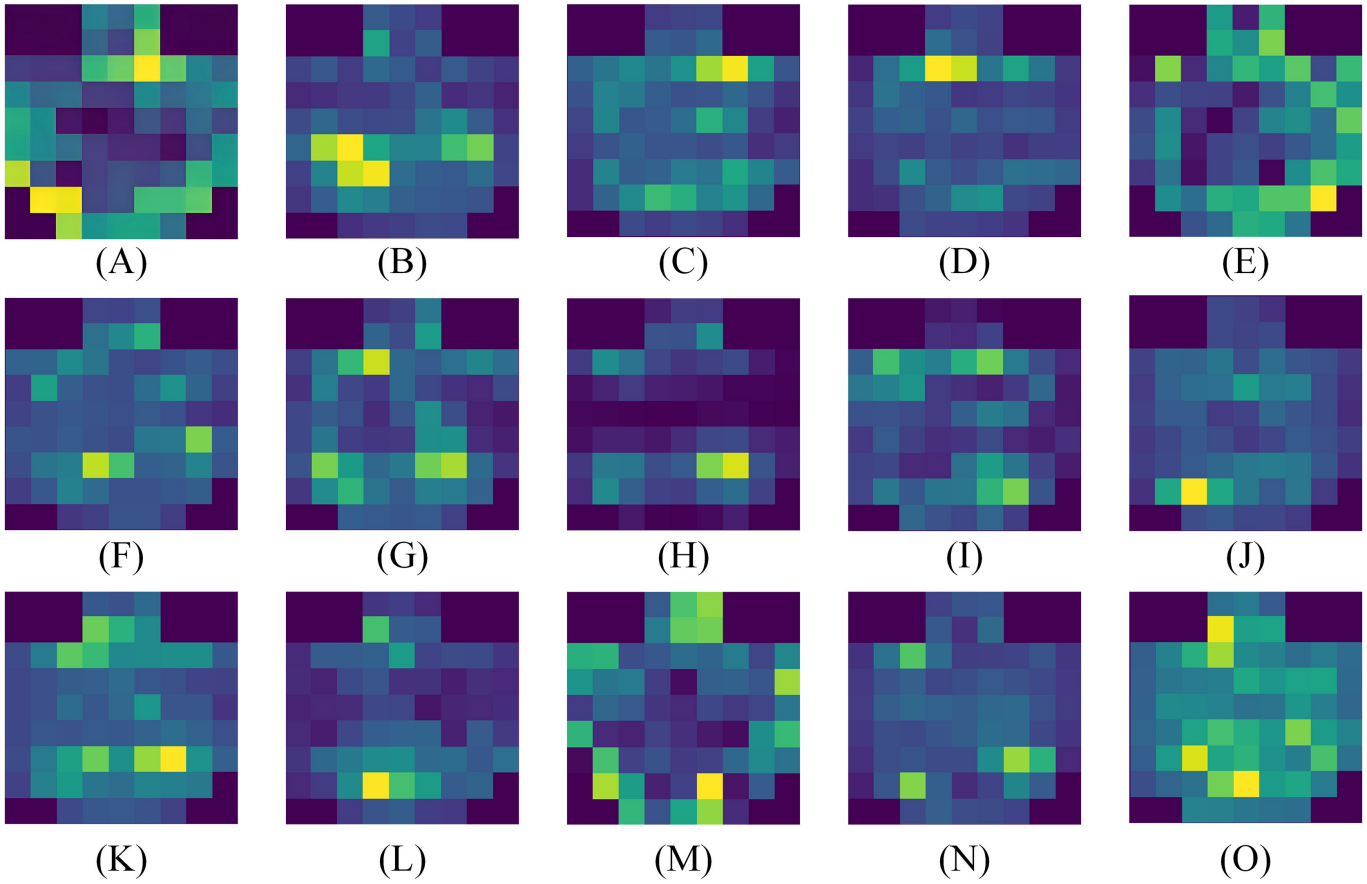
Average visualization results of each subject in the SEED dataset. **(A**–**O)** correspond to 15 subjects, respectively.

Based on the experimental results in the previous section, it has been demonstrated that our attention module can filter out key channels and brain regions. However, further research is needed to identify the brain activation areas and electrode channels corresponding to different emotions. To this end, we utilize the feature maps to analyze the specific feature patterns corresponding to negative, neutral, and positive emotions. Figures 8B, D, 9B, D, F, respectively, visualize the average emotional feature patterns of all subjects in the DEAP and SEED datasets. We find that there are indeed specific neural features for different emotional states. When a certain emotion is activated, the attention weights are mainly distributed in the prefrontal cortex (around channels FP1, FPz, FP2, AF3, AF4, F1, FZ, F2) and the lateral temporal lobes (around T7, T8, TP7, TP8, P7, P8, PO7, PO8). This is consistent with the conclusions obtained in the field of neuroscience (Allen et al., 2018; Etkin et al., 2015; Pozzi et al., 2021).

Specifically, when positive emotions are generated, channels located in the lateral temporal lobe receive significantly higher attention weights compared to neutral and negative emotions, followed by an increase in the frontal lobe. when negative emotions are generated, there is higher weight in the frontal lobe, while the electrode channels in the temporal lobe exhibit lower weight. From the visual results, neutral emotions are similar to negative emotions. The frontal lobe area is not given a particularly high attention weight value, but the activation degree of the parietal lobe and occipital lobe is slightly increased for neutral emotions. Moreover, our experimental results clearly indicate the presence of lateralization between brain regions, further supporting the idea that specific brain regions are more inclined to handle certain tasks or functions, rather than processing evenly across both sides of the brain. The activation states of brain regions corresponding to different emotions are also consistent with previous research findings (Ding et al., 2022; Gong et al., 2023).

To observe the differences between subjects more clearly, we also visualized the average emotional feature patterns of each subject in the SEED dataset, as shown in Figure 9, The figure not only confirms the aforementioned conclusions but also reveals that lateralization is not present in every individual. For some subjects, the lateralization in brain regions is not particularly pronounced.

## 5 Discussion

In this paper, we proposed HASTF to extract spatio-temporal features from EEG for emotion recognition. We validated our approach using two publicly available datasets. HASTF outperformed many state-of-the-art models and achieved excellent performance on both datasets. This indicates that our model can maintain high accuracy in recognizing emotions induced by both music videos (DEAP) and movie clips (SEED). Some models, such as Li et al. (2023) and Yang et al. (2024), excel in one induction method but falter in another. This also demonstrates that HASTF not only has excellent recognition performance but also possesses universality and strong robustness, performing stably across different datasets and experimental environments.In general, HASTF has the ability to capture both spatial and temporal dependencies, has the potential for application in online detection systems. In future online detection systems, we will further optimize computational efficiency to reduce the latency of deployment.

Our model introduces a novel two-part structure comprising a spatial attention feature extractor and a temporal attention feature extractor. Different from traditional GCN-based (Lin et al., 2023; Li et al., 2023; Jin et al., 2024) or convolution-based approaches (Ju et al., 2024), this spatial attention extractor employs a parameterless attention module. It perfectly matches 3D features and can adaptively select crucial electrode channels. As shown in Section 4.4, this module not only optimizes feature extraction but also eliminates the need for additional parameters, making the model more efficient and versatile across varied subjects. Furthermore, by incorporating skip connections, we mitigate information loss and ensure better integration of shallow and deep features throughout the iterative process. Tables 1, 2 shows that our spatial attention feature extractor surpasses existing methods in capturing key spatial features. Similarly, our temporal attention feature extractor leverages a multi-head self-attention mechanism to capture richer temporal features than previously used methods like RNN and TCN (Shen et al., 2020; Xiao et al., 2022; Tao et al., 2020; Yang et al., 2024). Ablation experiments confirm that the combination of these two attention mechanisms leads to state-of-the-art performance, underscoring the effectiveness of our spatio-temporal fusion approach. This integrated model advances EEG-based emotion recognition while providing a more adaptable and efficient framework for complex spatio-temporal data.

Finally, we also used the superior performance of HASTF to conduct in-depth analysis of EEG signals to capture the traces of emotions in various brain areas. As shown in Figures 7, 8, the lateral brain regions, especially the frontal and temporal lobes, are closely link to emotions. Specifically, positive emotions correspond to the temporal lobe, while negative emotions are associated with the frontal lobe. For neutral emotions, attention increased in occipital and parietal regions. However, we also observed significant individual differences in the model across subjects, as shown in Figure 9, manifested in different degrees and locations of lateralization. This difference suggests that cross-subject generalization remains a challenge. Although our attention mechanism can enhance attention to specific channels, different subjects have different weight assignments to the same channel, which complicates the calculation of channel weights across subjects. Therefore, future work will focus on how to design more flexible attention modules that can extract appropriate spatial and temporal features for different individuals to improve the performance of cross-subject emotion recognition. This will be the focus of our next research.

## 6 Conclusion

In this paper, we propose a hybrid attention fusion model capable of extracting spatio-temporal features for emotion recognition. The model uses spatial attention feature extractor to select crucial channels, extracting spatial features, integrates temporal attention feature extractor to select temporal features. Extensive experiments on two public datasets show that HASTF achieves superior performance than other methods. We also explore the role and interpretability of the spatial attention mechanism, visualizing the adaptive crucial channels and brain regions. Finally, we employ this model to capture the traces of emotions in the brain corresponding to different emotions and different subjects, proving that emotion-related neural patterns do exist, which is consistent with conclusions in the field of neurology. This research has promoted the progress of aBCI, which can be combined and applied to fields such as Emotion-Enhanced BCI, Human-Robot Interaction, Mental Health Detection and Autonomous driving, making our lives more convenient and intelligent.

## Data Availability

The dataset used in this study can be found at: DEAP dataset (https://www.eecs.qmul.ac.uk/mmv/datasets/deap/), SEED dataset (https://bcmi.sjtu.edu.cn/home/seed/).
